# Integrated Telehealth and Telecare for Monitoring Frail Elderly with Chronic Disease

**DOI:** 10.1089/tmj.2017.0322

**Published:** 2018-12-01

**Authors:** Hulya Gokalp, Joost de Folter, Vivek Verma, Joanna Fursse, Russell Jones, Malcolm Clarke

**Affiliations:** ^1^ Computer Science Department, Brunel University, Uxbridge, United Kingdom.; ^2^ Chorleywood Health Centre, Chorleywood, United Kingdom.

**Keywords:** *ageing*, *assistive technology*, *chronic disease*, *decision making*, *habits*, *integrated care*, *pervasive care*, *telehealth*, *e-health*, *telecare*, *telemetry*, *elderly care*, *activities of daily living*, *well-being*, *telemedicine*

## Abstract

***Objective:*** *To investigate the potential of an integrated care system that acquires vital clinical signs and habits data to support independent living for elderly people with chronic disease.*

***Materials and Methods:*** *We developed an IEEE 11073 standards-based telemonitoring platform for monitoring vital signs and activity data of elderly living alone in their home. The platform has important features for monitoring the elderly: unobtrusive, simple, elderly-friendly, plug and play interoperable, and self-integration of sensors. Thirty-six (36) patients in a primary care practice in the United Kingdom (mean [standard deviation] age, 82 [10] years) with congestive heart failure (CHF) or chronic obstructive pulmonary disease (COPD) were provided with clinical sensors to measure the vital signs for their disease (blood pressure [BP] and weight for CHF, and oxygen saturation for COPD) and one passive infrared (PIR) motion sensor and/or a chair/bed sensor were installed in a patient's home to obtain their activity data. The patients were asked to take one measurement each day of their vital signs in the morning before breakfast. All data were automatically transmitted wirelessly to the remote server and displayed on a clinical portal for clinicians to monitor each patient. An alert algorithm detected outliers in the data and indicated alerts on the portal. Patient data have been analyzed retrospectively following hospital admission, emergency room visit or death, to determine whether the data could predict the event.*

***Results:*** *Data of patients who were monitored for a long period and had interventions were analyzed to identify useful parameters and develop algorithms to define alert rules. Twenty of the 36 participants had a clinical referral during the time of monitoring; 16 of them received some type of intervention. The most common reason for intervention was due to low oxygen levels for patients with COPD and high BP levels for CHF. Activity data were found to contain information on the well-being of patients, in particular for those with COPD. During exacerbation the activity level from PIR sensors increased slightly, and there was a decrease in bed occupancy. One subject with CHF who felt unwell spent most of the day in the bedroom.*

***Conclusions:*** *Our results suggest that integrated care monitoring technologies have a potential for providing improved care and can have positive impact on well-being of the elderly by enabling timely intervention. Long-term BP and pulse oximetry data could indicate exacerbation and lead to effective intervention; physical activity data provided important information on the well-being of patients. However, there remains a need for better understanding of long-term variations in vital signs and activity data to establish intervention protocols for improved disease management.*

## Background

The increase of the aging population presents challenges to social care and healthcare, in particular, the prevalence of chronic disease among the elderly and need for long-term management is increasing healthcare costs. In addition, the level of independence of the elderly may fall due to disability resulting from aging, a disease, or cognitive ability,^1^ all of which may undermine their autonomy and make them dependent on care providers and social services. Combined with decrease in young population in developed countries, a need for new care plans that require less human resource and that combine health and social care services has emerged. Telemonitoring technologies have been considered for care delivery in the elderly with a high level of need and who require long-term care,^2^ thereby extending the period of independent living through timely intervention when deterioration in their well-being is detected. Ideally, timely intervention would result in averting hospitalization, speedy recovery, improved outcome and quality of life, and decrease in cost of treatment.^3^

The potential of telemonitoring technologies to improve management of chronic diseases and reduce cost to the healthcare system has been extensively researched over the last three decades.^3–6^ Most of these studies have focused on congestive heart failure (CHF), diabetes, hypertension, stroke, and chronic obstructive pulmonary disease (COPD), as timely intervention for these diseases can significantly improve the outcome of intervention and reduce cost of care.^3,7^ Vital signs that have been monitored include electrocardiogram (ECG), blood pressure (BP), blood glucose, pulse, oxygen saturation (SpO_2_), weight, and body temperature.^8^ Most studies have reported positive effects of telemonitoring.^9^

Changes in daily activity level and habits can provide vital information in relation to functional capabilities, deterioration in well-being, progress of an existing chronic disease, and loss of autonomy.^10^ Acknowledging this, over the last two decades, many studies have been conducted to investigate the potential of telemonitoring activity profiles of subjects to detect deterioration in their well-being and changes in lifestyle.^11,12^ These studies did not necessarily target the elderly with chronic disease; they reported results of technology development and evaluation of technological feasibility, and only a few of the studies associated changes detected in activity profiles with well-being of the subjects being monitored. Results that associated changes in activity and well-being included increased bathroom visits due to urinary tract infection and increased level of nocturnal activities was thought to be sign of deteriorating cognitive abilities. Approaches and sensors used to acquire activity data varied, and included activity-log records, passive infrared (PIR) motion sensors, electricity used by appliances and accelerometer-based wearable sensors. Parameters monitored included activity level, bed restlessness, bathroom visits, forgotten stove burner, body movements, and posture (walking, running, standing, and fall).^11^

Few studies have monitored physiological parameters together with activity data.^11^ Most of the projects were restricted to using volunteers to test the feasibility of their systems; only a few of them involved elderly with chronic disease(s) with the aim to predict key medical events that required intervention or changes in habits profile that were associated with deterioration in well-being of elderly with CHF.^13^ Only a few have investigated the association between changes in clinical and activity data, with results being encouraging for the relevance of monitoring activity data of subjects with chronic disease(s).^14^

Acknowledging the growing demand for independent living among elderly in developed countries, a research project, entitled Integrated Network for completely assisted Senior citizen's Autonomy (inCASA) was developed to demonstrate the concept of integrated health and social services for the frail elderly living alone.^15^ Since there were no commercial systems available to support integrated telehealth and telecare, an integrated platform for telemonitoring of vital signs and habits data was developed for the United Kingdom pilot. The platform was used to manage 36 frail elderly who were registered with Chorleywood Health Centre (UK), received care from social services, had a chronic disease, and were living alone. The telemonitoring system was purpose designed for the elderly, having several important features, some of which are unique to the system:

- IEEE 11073 standard-based semantically interoperable platform
- Nonobtrusive
- Simple to use
- Plug and play installation; self-integration of sensors to the system
- Monitors both activity and physiological data
- Online analysis of data and alert

This article presents results from the U.K. pilot where habits and vital signs of 36 frail elderly with chronic disease(s) were monitored. Our aim was to investigate:

- Feasibility of the concept of integrating health and social care on a single platform.
- Habits profiles of elderly and rules to notify professionals when there is deviation from normal patterns.
- Whether change in habits profile is associated with patient's well-being.
- Advantages of sharing and exchanging information between the primary care and social services.

Following a brief description of the study design and monitoring system, we present results of the data analysis and discuss findings.

## Materials and Methods

### Participant Identification and Recruitment

Subjects for the pilot were selected from patients registered with Chorleywood Health Centre, United Kingdom, using the following criteria:

- Over the age of 65 years
- Have at least one chronic disease
- Living alone
- Determined to be “Frail” as defined by the Edmonton Frailty Score^15^
- Had an unplanned hospital admission in the past 6 months or two in the past 12 months.

One hundred five (*n* = 105) patients were identified as meeting the inclusion criteria, and were informed of the study and invited to participate. Ethical approval for the study was gained from the local research ethics committee.

A total of 44 patients initially gave informed consent to participate, and 36 were recruited into the study from October 2012 onward. After the end of the pilot phase on May 31, 2013, some remained in the service till March 2014, which enabled us to obtain monitoring data for longer than a year.

The service team was made up of clinical nurses, general practitioners, nonclinical researchers, social service workers, administrators, and technical support; the service provided guidelines for self-management and the communication channel (mainly phone) between patients and their nurse care managers.

### Telemonitoring System—Sensors; Home Gateway; Remote Server; Clinician Portal

**Fig. 1.**
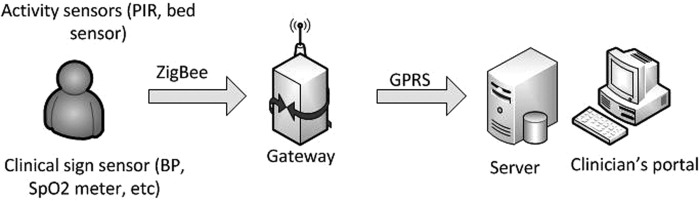
inCASA monitoring platform.

**Fig. 2.**
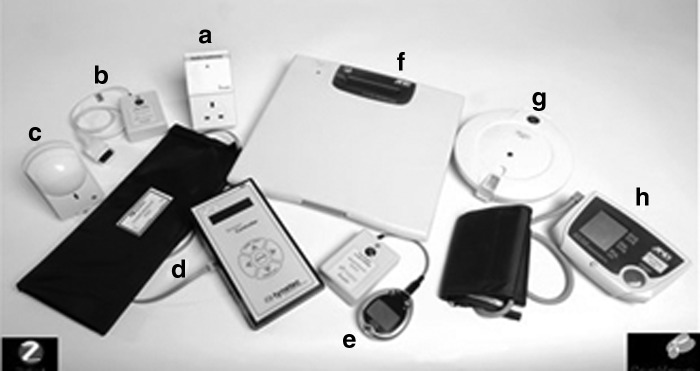
Gateway and sensors used: **(a)** the gateway, **(b)** pulse oximeter, **(c)** PIR motion sensor, **(d)** bed sensor, **(e)** glucose meter, **(f)** weight scale, **(g)** medication dispenser, **(h)** BP meter. BP, blood pressure; PIR, passive infrared.

The Home Monitoring Platform was designed and deployed to participant's house.^16^ The platform comprised of sensors to acquire patient's habits and clinical data, a home gateway, a remote server to store patient data, and a clinician portal to view and manage patient data and records (*Fig. 1*). The platform used a standards based approach for data communication that enabled many different types of devices, habits, and health, to be deployed to patients with co-morbidities. The IEEE 11073 medical device standards^17^ were used for communication from the sensor to the gateway; IHE PCD-01,^18^ a profile of HL7^19^ was used for data communication from the gateway to the server. All data are automatically transmitted wirelessly from the sensor devices to the home gateway using the ZigBee Healthcare Profile,^20^ and then wirelessly to the remote server using GPRS. The activity and clinical sensors (*Fig. 2*) were obtained off-the-shelf and modified to take our IEEE 11073 radio modules to allow wireless data transmission to the gateway.

### The Gateway

The gateway was designed to be simple, unobtrusive, and self-contained so that it required no configuration for installation and being based on cell-phone technology (GPRS) avoided the need for patients to have existing internet connectivity or landline. It had no user interface other than an LED to indicate connection to the server, and its installation was as simple as “plug it into a mains socket and watch for the green light indicating connection to the remote server.” As there was no user interface, sensors could be installed anywhere in the home. The installation of the telemonitoring equipment was carried out mostly by the nurses. Devices were located by taking into account both the preference of the patient and the quality of wireless connections.

Patients were given training during the installation, which included how to operate their clinical sensor(s) and observe the light on each device to confirm successful data transmission. They were also given the contact numbers of the clinical team, whom they could contact in case of a concern.

Data were transmitted from the gateway to the clinical server over a secure private mobile network; the clinical server was located in the secure data center. Patients were assured that data would be managed securely and kept private, and any data would be published anonymously.

### Sensors and Parameters Extracted

The first generation gateway could support up to 3 sensors connected concurrently, and the second generation gateway could support up to 10 sensors. In general, three sensors were installed in the home of each patient, depending on the disease. Patients with CHF were given a weight scale and a BP meter, and those with COPD were given a pulse oximeter (*Table 1*). All patients were given a PIR motion sensor in the living room, and those with a single health device were given a bed or chair occupancy sensor.

**Table 1.** Sensors Deployed

SENSOR	REASON	DATA COLLECTION FREQUENCY	MONITORING FOR
BP	BP in CHF	Daily	Exceed defined target >140/80 mmHg
SpO_2_	Oxygen saturation in COPD	Daily	Exceed defined target <85%
Weight	Fluid retention in CHF	Daily	Change of >1 kg in 24 h or 1.4 kg over 3 days
Motion sensor	Habits monitoring	Continuous	Movement variance from normal
Bed sensor	Habits monitoring	Continuous	Unusual time for bed occupancy; number of times out of bed during night
Chair sensor	Habits monitoring	Continuous	Unusual time in chair; excessive time in chair

BP, blood pressure; CHF, congestive heart failure; COPD, chronic obstructive pulmonary disease.

All the data from the sensors were sent automatically to the gateway and from the gateway to the remote server without user intervention, where they were used by the clinical team for management of the patient. Automatic transmission of data eliminated reporting bias of manual entry or confirmation,^21^ and was a very useful feature for the elderly due to the likelihood of physical and/or intellectual limitation.^1,22^ An alert algorithm, normally based on default thresholds as shown in *Table 1*, or customized limits, was applied to all incoming data to provide visual alerts for high BP, low SpO_2_, or significant change in weight on the clinical portal.

### Clinical Sensors

All the sensors were clinically validated, and were modified to take our ZigBee radio module to allow wireless data transmission to the gateway and then on to the remote server. Patients were instructed to take at least one measurement each day, where possible first thing in the morning before breakfast. With the BP meter and pulse oximeter, they were instructed to take their measurement after sitting quietly for 5 min and while their arm was resting on a table or the armrest of a chair.^22^

The BP meter was an upper arm cuff meter and patients were instructed to use it with the upper arm leveled with the heart.^22^ We chose a finger Pulse oximeter that provided a noninvasive estimation of arterial hemoglobin oxygen saturation (SpO_2_).

Occasionally patients took two or three clinical measurements on a single day. With SpO_2_ the higher reading was chosen as the reading for that day, as this approach is used by clinicians^23^; the median value of multiple readings on a day was used as the representative value for BP and weight readings.

### Habits Sensors

Two types of sensor were deployed to patients' homes to monitor habits: (1) PIR motion sensors (*Fig. 2c*) to detect movement in a location and (2) pressure sensors (*Fig. 2d*) to detect bed or chair occupancy. Our aim was to define a daily habits profile for the elderly person in their home to determine when there was deviation that might be indicative of change of well-being.

#### PIR sensors

The location of the PIR sensor was determined so as to capture and profile important and relevant daily activities. The sensors were typically located in the living room in a position to capture the significant movements within the home, such as from living room to/from the kitchen, bathroom, or bedroom, but not to capture movements while sitting in the chair or sofa.

#### Bed/chair occupancy sensors

These sensors were calibrated pressure sensors located underneath the mattress or the chair cushion and were configured to send a message for both “usage started” and “usage ended.” To avoid glitches in the sensor data, a change in state of usage message was only sent after the sensor had remained in its new state for 30 s.

A few patients asked for their chair-sensor to be removed as they found it uncomfortable; and a few of the sensors were found to be sensitive to changes in the room temperature and gave unreliable data. About 6 months into the monitoring period, the bed/chair sensors were replaced with PIR sensors in the bedroom due to comfort or reliability issues.

### Parameters Extracted from Activity Data

We analyzed the data to define a normal profile for each of the parameters, typically formed from the moving average of data (defined for each parameter), and from this we determined deviations from the normal profile to investigate whether deviations are associated with the well-being of the patient. Some of the algorithms and parameters were used to provide alerts on the clinical portal; others were used for retrospective analysis. Following a clinical intervention, we retrospectively analyzed the data to identify patterns or parameters that might predict the oncoming event. The following parameters were derived from the habits sensor data:

- Number of sensor events in a given period
- Mean of hourly movement counts
- Time of first movement in early morning and last movement in the evening
- Time to next sensor event
- Bed/chair occupancy in a given period.

The parameters were derived as follows:

#### Number of movements detected by PIR sensors and usage triggers in different time periods

The number of movements detected in each hour was counted. These were accumulated to determine the number for different time periods in the day and for the whole day. Similarly, the number of usage triggers from usage sensors was counted and accumulated. We used the variation from the normal value for the whole day from both PIR and bed/chair sensors to raise alerts on the clinical portal. We found the binning period of 1 h sufficiently short to determine the times of activities, but sufficiently long to filter out short-term daily variations in the times of activities.

#### Mean of hourly movement count

**Fig. 3.**
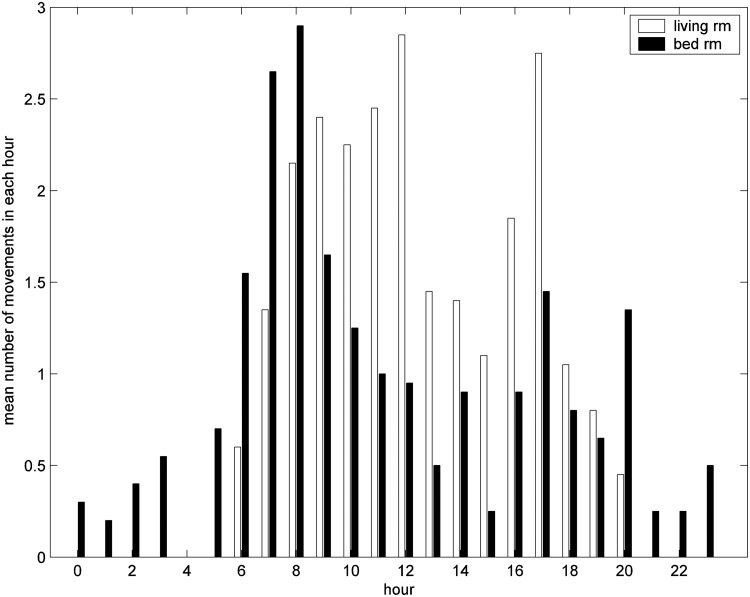
Mean number of movements detected by motion sensors per hour over the period between day 251 and 270 for living room (white) and bedroom (black).

Data from a PIR sensor across 20 days were used to determine the activity profile of a subject across the day (e.g., *Fig. 3*). Depending on the location of the PIR motion sensor, it would be possible to estimate the time for: getting out of bed, breakfast, lunch, dinner, and going to bed. For example, from *Figure 3*, we could infer that the subject got up at around 7 am, the high level activity around 8 am might correspond to breakfast, at around 5 pm to dinner, and the subject leaving the living room at 8 pm was going to bed.

#### Time for first movement in early morning and last movement in the evening

**Fig. 4.**
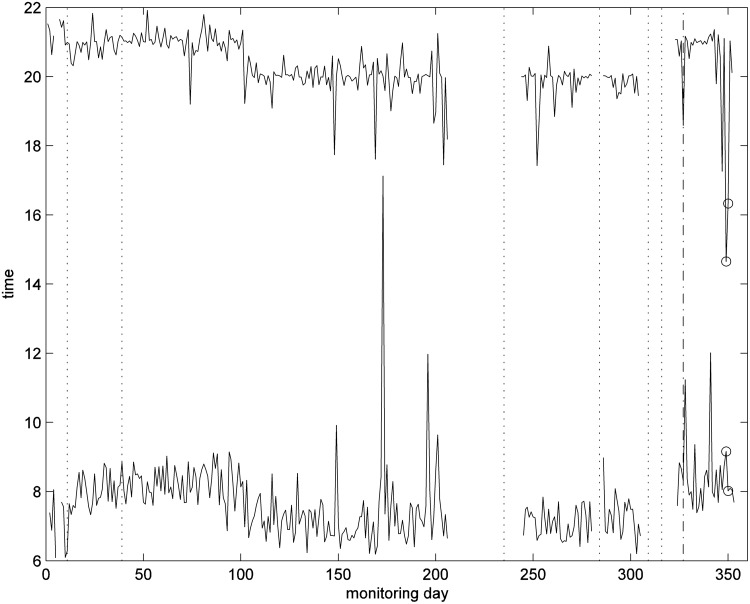
Times of first movement after 5 am and last movement before midnight in the living room.

Observing the mean of the number of movements in each hour in *Figure 3* highlights that this patient has a clear “bed time” and “wake-up time” routine; they get up between 4:00 and 6:00 and go to bed by 22:00. Using this knowledge and a simple algorithm, times of first movement in the morning and last movement in the evening can be estimated, and can be used as an estimate of bedtime routine, in particular, when no bed sensor is used, as in *Figure 4*.

#### Time to next move: time to next sensor event

We obtained the time intervals between consecutive sensor events; this gave us a time series of time intervals between consecutive events. We then determined the 90th quantile and median value of the time interval for days, where possible, with 30 movements or more.

#### Bed/chair occupancy

The bed/chair pressure sensor provides a time stamped event to indicate a change in state of occupancy. Using times of consecutive “usage started” and “usage ended,” we could calculate the length of occupancy for each usage, and then total bed/chair occupancy in a day by accumulating the individual occupancies.

### Raising Alerts from the Data

A simple algorithm was implemented to detect deviations from the norm for the habits data. For the clinical data, two types of thresholds were used: an absolute threshold taken from the clinical assessment protocols given in *Table 1*; and subject specific thresholds (mean ±2 standard deviation [SD]).

On the presence of an alert on the portal, further steps were taken: in the case of an activity alert the nurse would contact the patient to determine the reason for the alert, and if the alert persisted, a visit to the patient was planned. If the nurse considered that a change in treatment or medication was required, they would refer the patient to the doctor.

Initially alerts were generated from the activity data by dividing a day into four periods: (1) 00:00–06:00, (2) 06:00–12:00, (3) 12:00–18:00, and (4) 18:00–24:00. The mean and SD of the number of sensor events for each period was determined by using a moving window of 15 days. Period-specific thresholds were calculated as mean ± 2SD. If the number of movements in a period fell outside the threshold values for that period, a red flag was shown on the clinicians' portal. However, after 6 months, the four time periods were deemed to be giving rise to too many false alarms; for example, the absence of a patient for part of a time period could easily result in an underactivity alert, or a visitor in the afternoon to an overactivity alert for that time period. From our experience and discussion with the clinical team, three time periods were found to be more relevant to well-being of a subject: (1) all-day (mid-night to mid-night), (2) night-time (22:00–06:00), and (3) morning (06:00–10:00). Instead of generating alerts for all time periods, we decided to generate alerts on the portal only for all-day.

### Clinical Portal

A clinical portal was developed to (1) visualize patients' data and alerts, (2) allow the clinician to view, manage patients' data, and edit patient records for the project, and (3) allow the research team to download the patients' data.

Alerts were displayed on the clinical portal to notify (draw the attention of) the clinicians to patients that may require intervention. The clinicians' portal was reviewed daily by a nurse to determine whether alerts had occurred and intervention might be required and to examine the data of specific patients to monitor progress (e.g., after change of treatment).

## Results

Thirty-six (*n* = 36) patients were enrolled in the service (mean age 82 years [SD = 10], 38% male, 56% average frail, and 27% very frail). The majority of the patients enrolled in the study were not familiar with new technologies. Acceptance of habits monitoring was an issue for about 15% of the patients for a number of reasons, including intrusiveness of the technology; did not want stigmatization of being “frail”; and did not feel that the technology was for them, as they did not consider themselves as frail. Generally most did not give a specific reason for declining participation, stating only that “they did not want to.”

Compliance rate with daily readings of BP and SpO_2_ was generally over 60%. Only a few patients had low compliance; two with CHF were very frail and only occasionally would weigh themselves due to safety concerns of standing on the weigh scales. One patient took only 17 BP measurements over 75 days and one patient took only 3 SpO_2_ readings over 110 days. We did not investigate the reasons for low compliance rate for BP and SpO_2_.

Fifty-five percent (20) of patients were referred for investigation during the time that they were being monitored; 44% (16) received some type of intervention. The most common reason for intervention was due to low oxygen levels for patients with COPD; these patients were referred to community pulmonary services. We illustrate our preliminary results with four case studies.

### Patient 1—Chf and Associated Hypertension

This patient had CHF and associated hypertension and was monitored for 353 days. A PIR sensor in the living room, a chair sensor, and BP meter were deployed to this subject. However, the subject asked for the chair-sensor to be removed due to discomfort issues. Later in the monitoring period (day 244), a PIR sensor was installed in the bedroom.

**Fig. 5.**
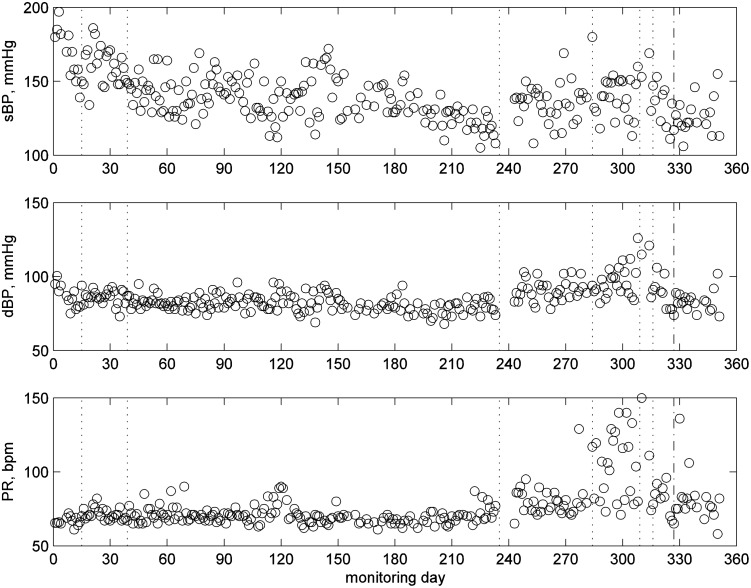
BP readings for patient 1—showing days with clinical concerns and medication change (vertical dotted lines) and nighttime event (dash and dot vertical line): systolic BP (top), diastolic BP (middle), and pulse rate (bottom).

*Figure 5* illustrates the BP readings. The patient measured the BP for 281 days of the 351 monitoring days. BP readings varied considerably over the monitoring period: they were mainly over 150 mmHg at the beginning of the monitoring, which lead to BP assessment and a medication change on day 15. The medication change helped to reduce the BP level. Around day 220, the BP value fell with the patient complaining of dizziness, which led to a further medication change on day 235. The subject complained of swollen ankles, which led to a further medication change on day 294, followed by another on day 309. The subject felt worse on day 316, and there was a nighttime event on day 327.

The variability of the systolic BP and pulse increased significantly from day 270 onward, and the patient was diagnosed with atrial fibrillation (AF) on day 284.

**Fig. 6.**
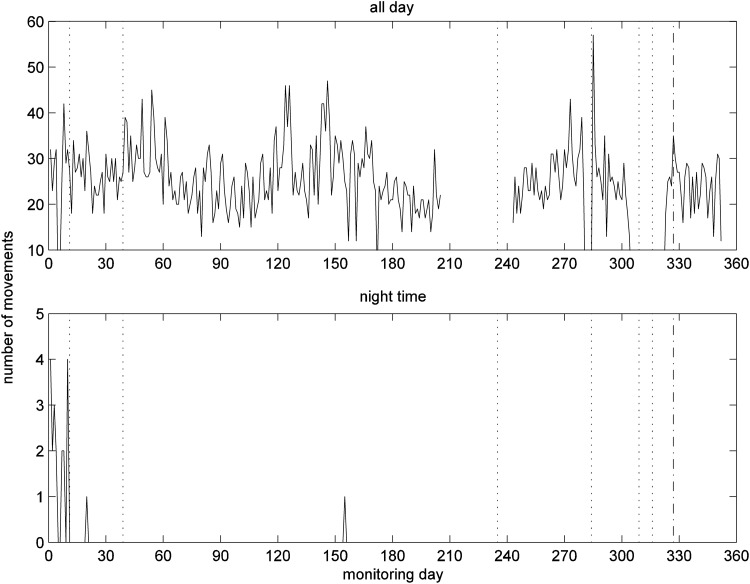
Number of movements detected by motion sensor in living room for whole day (top) and for nighttime (bottom).

*Figure 6* illustrates the number of movements detected by the PIR motion sensor in the living room for the whole day and the nighttime (22:00 pm–6:00 am) periods. The living room PIR event counts for the whole day exhibited a fluctuating trend with a period of about 90–120 days; however, we cannot offer any physiological explanation for this. Although there are other fluctuations, we cannot find any association with clinical events.

Nighttime activities resulted in an alert on day 15; when contacted the patient reported having a heavy cold and so was up and down all night. After this event, the nighttime activity level remained zero except for two occasions.

**Fig. 7.**
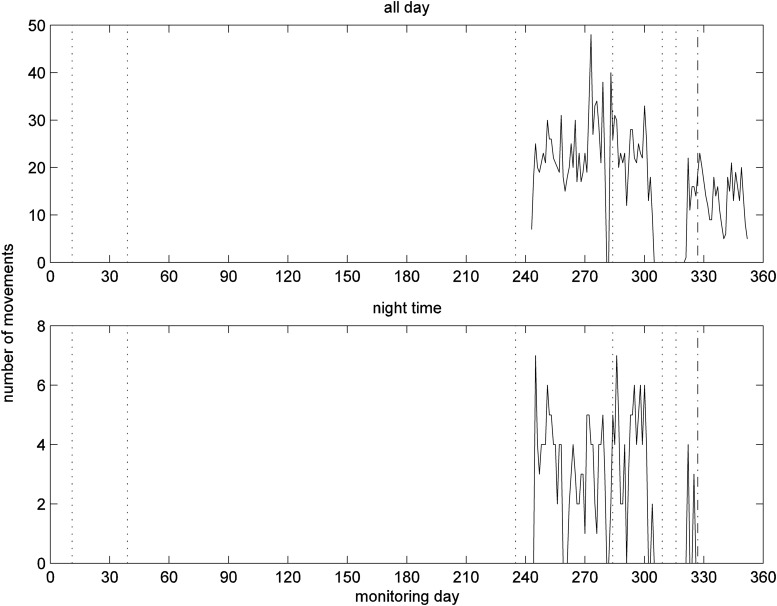
Number of movements detected by motion sensor in the bedroom for whole day (top) and for nighttime (bottom).

The results for the PIR sensor in the bedroom complement those of the living room and are given in *Figure 7*. After day 325, there is a slight drop in bedroom activity level for whole day; after day 327 no activity was detected during nighttime.

**Fig. 8.**
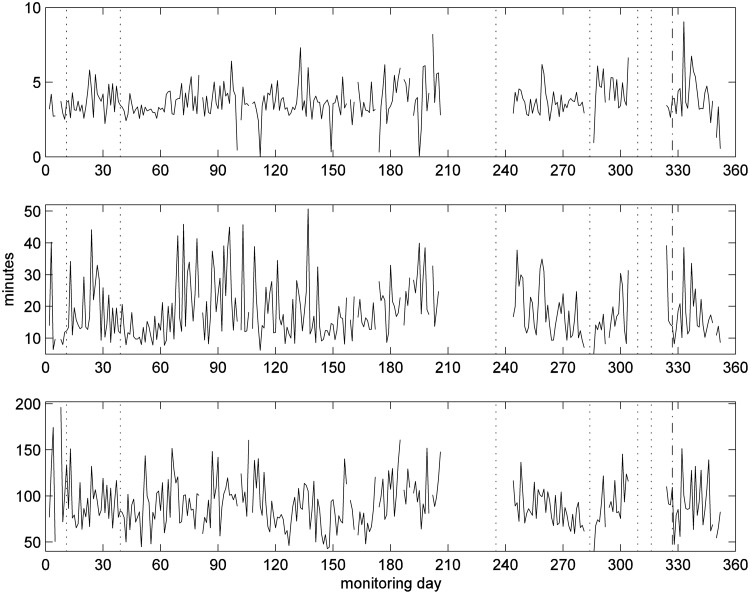
Quantiles of time-to-next-move for each day with daily move counts greater than 15: the 10th quantile (top), 50th quantile (middle) and 90th quantile (bottom).

We further analyzed the living room PIR data by calculating the time between successive events to determine the time to next move. We then ranked the time to next move values for the whole day (*Fig. 8*). Values of the 10th quantile could give information on the length of time small tasks took to perform, such as going to the kitchen/bathroom and coming back. Those for the 90% values will give information about the length of prolonged periods with no movement; the longer this value the less likely the patient would want to move. The values for the 50th percentile will indicate general tendency for time-to-next-move values. The value for the 50th quartile has episodes where the value increases, notably around day 20, between day 85 and 140, and day 340.

*Figure 3* illustrates the combination of the average number of PIR events for each hour for the living room (white bar) and bedroom (black bar) for the days from 251 to 270 to determine the pattern of behavior around the house throughout the day and night. The subject usually has no nighttime activity in the living room and has a very predictable pattern for first and last movements detected in the living room each day. This subject habitually first enters the living room at around 5:00 am and leaves before 22:00 pm.

We therefore observed the daily times for the first and last movements detected in the living room, as shown in *Figure 4*.

**Fig. 9.**
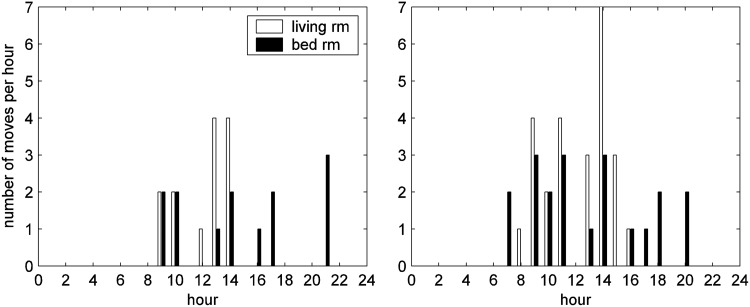
Number of movements detected by motion sensors per hour for living room (white) and bedroom (black), for day 349 (left) and day 350 (right).

To demonstrate, *Figure 9* shows the hourly activity graph for the living room and bedroom for day 349 (left) and day 350 (right). Each exhibits an unusually early last movement, with the subject last leaving the living room in the early afternoon (e.g., 14:00 day 349) and the bedroom activity confirming they spent the remainder of the day in the bedroom. There is a corresponding late first movement the next day in the living room (8:00). We therefore investigated the other days with unusually early last movement for the period with two PIR motion sensors (one in living room, the other in bedroom), and found that the patient spent the remainder of the day in the bedroom. In contrast, there was no significant difference (i.e., no indication of such a pattern) in the total daily and nighttime PIR event counts for the living room (*Fig. 9,* left) and bedroom (*Fig. 9,* right) to generate an alarm.

We observed an increase in the number of these incidences after day 300, and these correlated with the problems seen with their BP (*Fig. 5*) and diagnosis of AF, and would also be associated with the patient reporting on day 316 that they were feeling worse.

### Patient 2—Chf and Associated Hypertension

This patient had CHF and associated hypertension and was monitored for 495 days. At the start of the study, the patient was given a BP meter, and a PIR sensor (in the living room) and a bed sensor were deployed. Due to reliability issues, the bed sensor was removed and replaced by a PIR sensor in the bedroom on day 253.

**Fig. 10.**
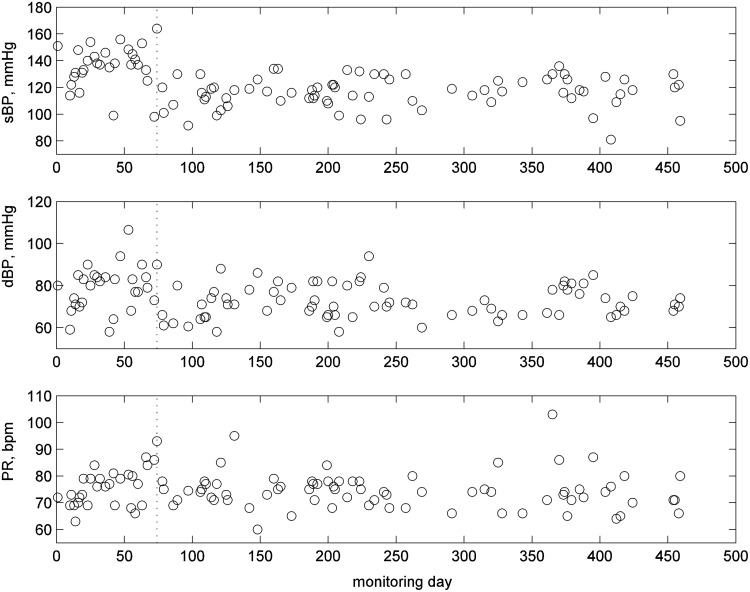
BP readings for patient 2: systolic BP (top), diastolic BP (middle), and pulse per second (bottom).

*Figure 10* shows the 105 BP readings that were taken by the patient during the monitoring period. Typical systolic BP was around 145 mmHg or higher for the first 70 days, which generated alerts on the clinical portal by being above the threshold of 140 mmHg and led to medication change on day 74. Systolic BP fell to around 120 mmHg after the medication change and remained below the threshold for the remainder of monitoring.

**Fig. 11.**
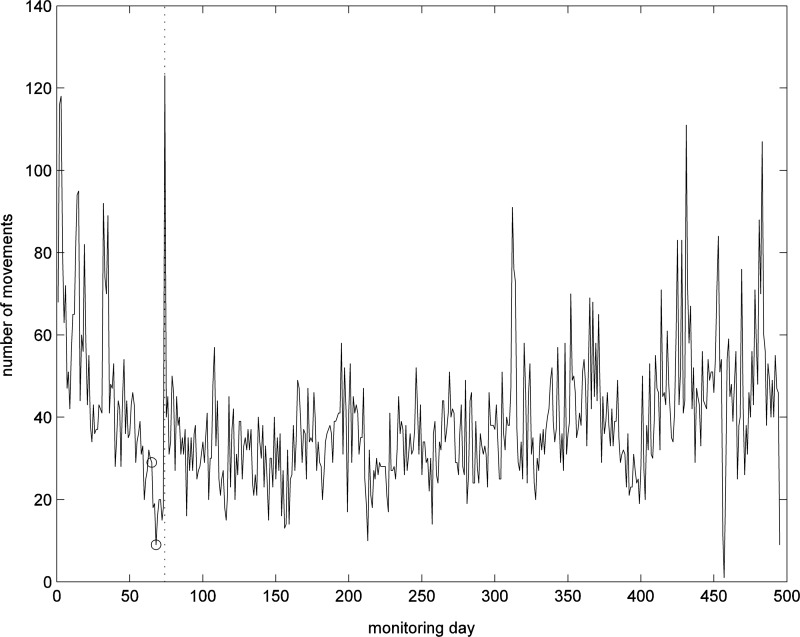
Number of movements detected by motion sensor in living room for whole day; clinical intervention marked by circle.

*Figure 11* shows the number of movements detected for whole day by the PIR sensor in the living room. The patient had several visits by the nurse during the first 40 days due to alerts on the portal for high BP, as seen by the increased number of movements on certain days. However, there was a trend of decreasing number of movements in daily PIR activities after day 50, which led to underactivity alerts on the portal. When the patient was contacted by phone on day 65, they said that they had stayed in bed longer after a recent fall. These incidents on day 65 and 68 are marked by a circle.

**Fig. 12.**
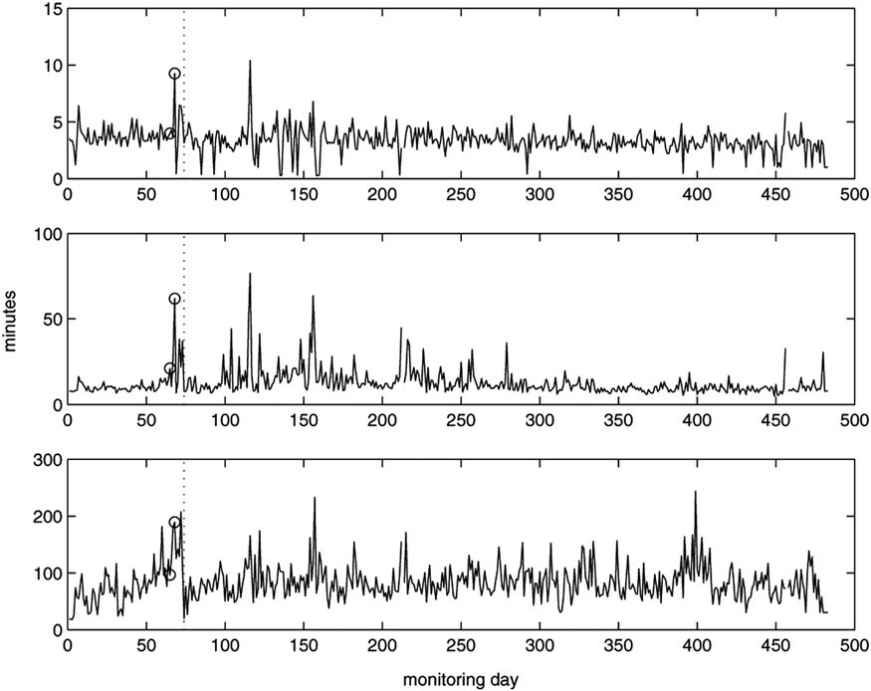
Quantiles of time-to-next-move for each day with daily move counts greater than 10: the 10th quantile (top), 50th quantile (middle), and 90th quantile (bottom); clinical intervention marked by circle.

A nurse visited the patient on day 74 and found that the patient had cellulitis. The visit by the nurse resulted in a peak in living room PIR activities. In contrast, the patient movements were becoming fewer after the fall event (*Fig. 11*) and the median values of time-to-next-move were increasing (*Fig. 12*). Note that the median values of time-to-next-move are less sensitive to visits.

Inspection of the median (50th quartile) values of time-to-next-move (*Fig. 12*) appears to indicate that the patient continued to have health issues until around day 300 at which time the value returned to one comparable to the beginning of the monitoring period when the patient was considered in good health. We have no clinical events to corroborate.

### Patient 3—Copd

**Fig. 13.**
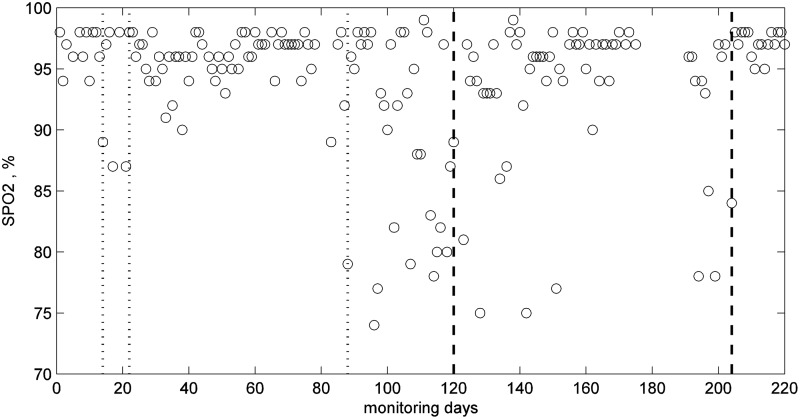
SpO_2_ readings for patient 3 indicating days of clinical concerns (vertical light dashed lines) and intervention (heavy dashed lines).

This patient had COPD and was monitored for 212 days. The patient was given a pulse oximeter, a PIR sensor in the living room, and a bed sensor. The bed sensor was only used for the first 62 days; therefore, no results are presented for this sensor. This patient had two major clinical events during the 212 days; hospital admission on day 120 for 3 days and a chest infection on day 204 (thick vertical dashed lines in *Fig. 13*). There are also notes indicating clinical concerns around day 22, 30, and 88 (light vertical dashed lines in *Fig. 13*). The patient, when contacted, did not report any change in condition on day 14 or 22; and believed that their breathing had improved around day 14. On day 88, the patient was diagnosed with a cold, and the condition continued to deteriorate until the patient was admitted to hospital on day 120. Our earlier work on analysis of daily readings of SpO_2_^23^ (*Fig. 13*) showed that the short-term and long-term trends and residuals closely followed the condition of the patient, that is, decreasing level in trends and increasing SD of residuals during periods of clinical events, and returning to their usual levels following the interventions.

**Fig. 14.**
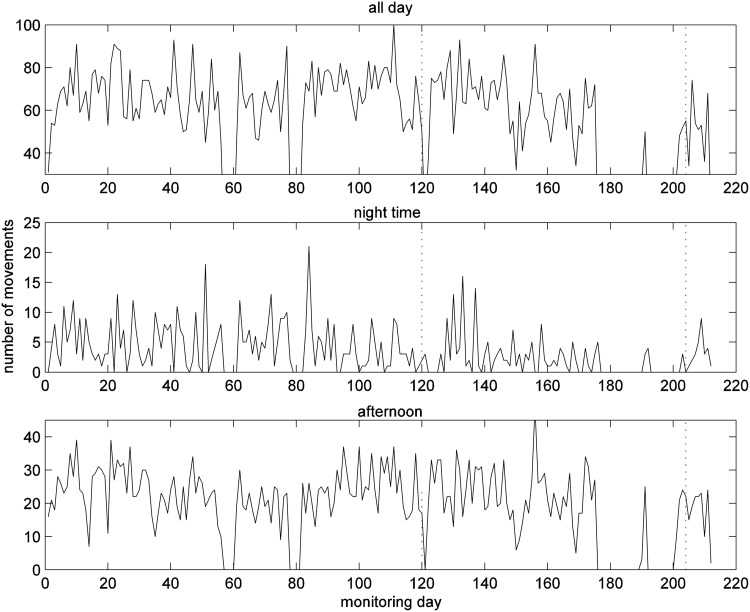
Number of movements detected by motion sensor in living room for whole day (top), nighttime 22.00–6.00 (middle), and afternoon 12.00–18.00 (bottom).

**Fig. 15.**
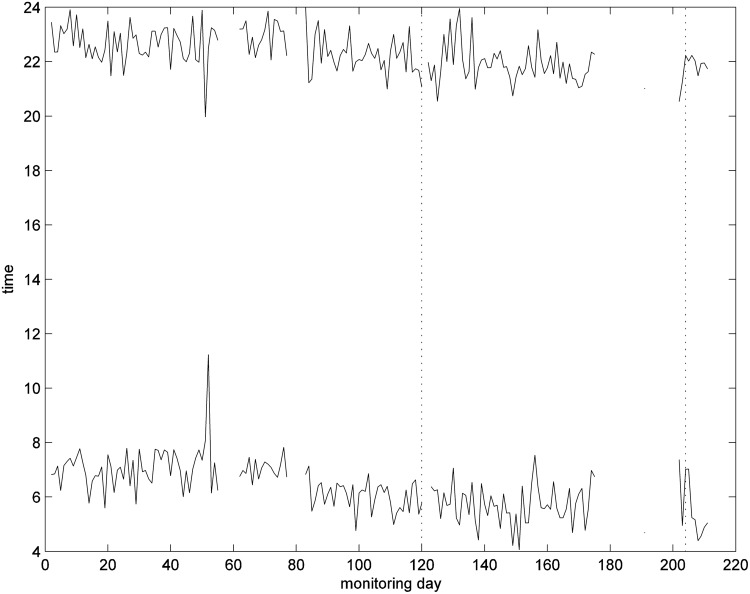
Times of first movement after 4 am and last movement before midnight in the living room.

The number of movements detected by the PIR motion sensor in the living room and the times of first and last detected movement in each day are given in *Figures 14* *and* *15* respectively. There is a slight increase in movement counts for the whole day between monitoring days 85 and 110, from about 60 movements to 70 movements (*Fig. 14*). The reason for this may be discomfort or that they had to pause to take a breath or were walking more slowly, both of which would have led to extra PIR detection events while they were walking through the detection zone. From around day 90 onward, more movements were detected in the afternoon. These increases in number of movements coincided with very low SpO_2_ levels. The patient also appears to be particularly restless on some nights (50, 85 and 130). This patient started to get up slightly earlier after day 80 (*Fig. 15*), which coincides with the summer daylight savings time change, and is not significant. There is 1 day (50) when the patient appears to have retired to bed earlier than usual.

**Fig. 16.**
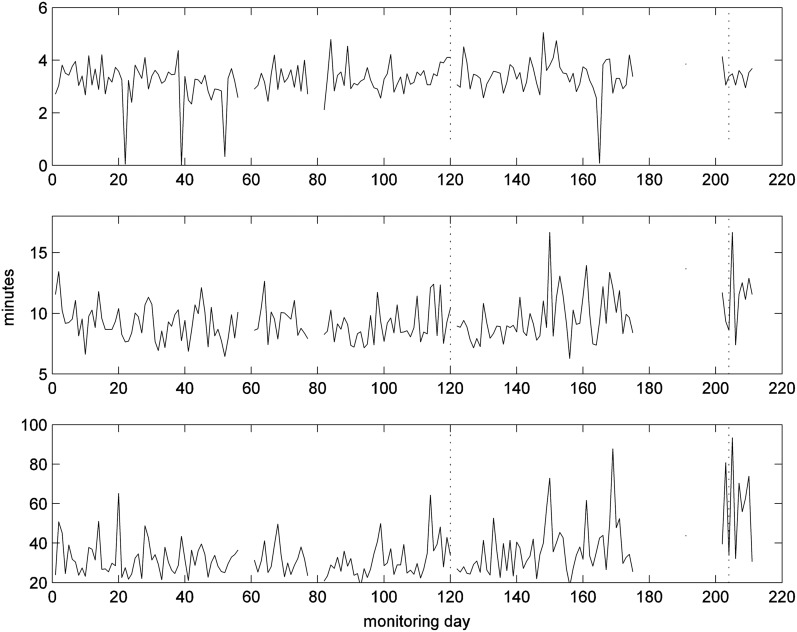
Quantiles of time-to-next-move for each day with daily move counts greater than 30: the 10th quantile (top), 50th quantile (middle), and 90th quantile (bottom).

*Figure 16* presents the 10th, 50th, and 90th quantiles for time-to-next-move for days with 30 movements or more. The values for the 90th quantile became slightly higher before day 120 and after day 200, that is, toward the hospitalization. The reason for this may be that the subject would tend to walk more slowly due to difficulty in walking during exacerbations (when SpO_2_ values are low)^21^ and/or was taking longer to complete a task.

### Patient 4—Copd

**Fig. 17.**
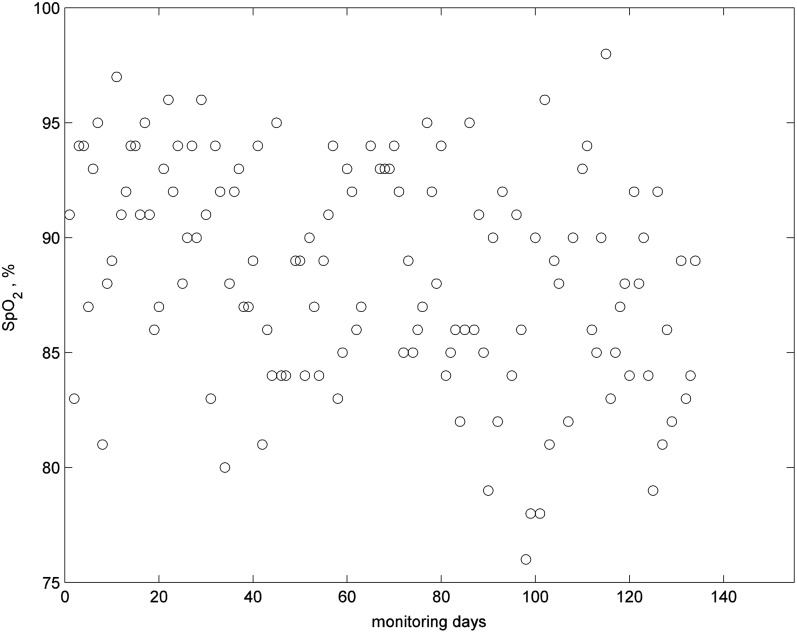
SpO_2_ readings for patient 4.

This patient had pulmonary fibrosis, and died at home on day 135. The patient was given a pulse oximeter, a PIR sensor in the living room, and a bed sensor. We had data from the bed sensor for almost the whole monitoring period, but data from PIR motion sensor for only the first 57 days; therefore, we do not present results from the motion sensor. *Figure 17* shows the SpO_2_ readings. The patient commenced oxygen therapy on day 34, and reported that therapy was helping. The long-term trend indicated a steady decline in the condition of the patient over the monitoring period.

**Fig. 18.**
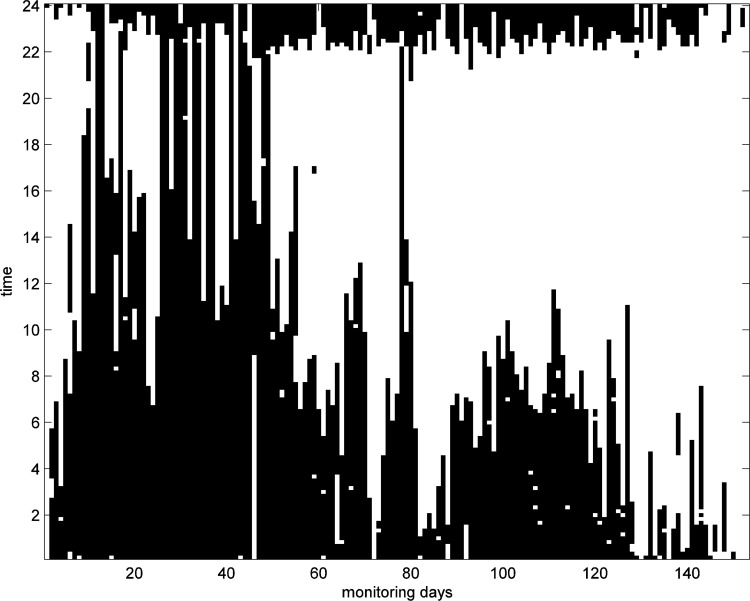
Times of bed occupancies as stacked vertical black bars for each day.

The bed data provided important information on the well-being of the patient. *Figure 18* shows the periods of bed occupancy over the monitoring period as stacked vertical lines. Midnight appears at the top and bottom of the *Figure 18*, with periods of bed occupancy in each day shown as black vertical lines.

*Figure 18* shows the bedtime routine and behavior over the period of monitoring. The patient retires to bed at a fairly constant time each day between 22:00 and 24:00; however, the time of waking varies and indicates a significant drop in bed occupancy between days 80 and 90 during an exacerbation, and day 125 onward as death approaches. This change may be due to being unable to sleep or experiencing difficulty with staying in the bed due to breathing difficulties.

**Fig. 19.**
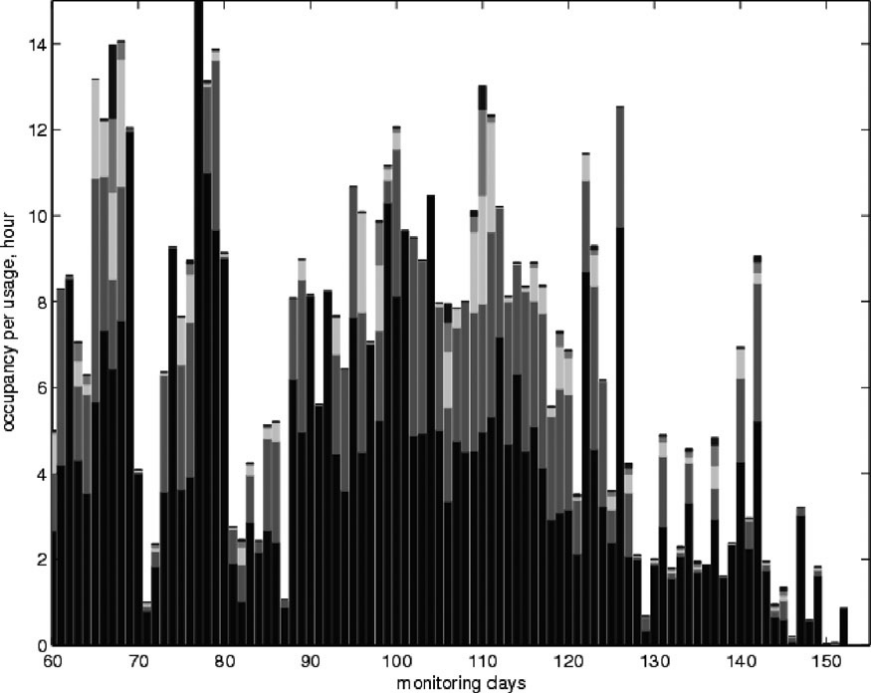
Ordered occupancies of the five longest occupancies each day for the last 90 days.

Close inspection of *Figure 18* shows that the sleep pattern is broken during each day. We therefore identify each period of bed occupancy during the day and order them according to decreasing length. *Figure 19* shows the stacked bar graph of the lengths of the five longest occupancies each day during the final 90 days. The five longest occupancies using a stacked bar graph (*Fig. 19*) can provide information on the length of each period of uninterrupted sleep, and thus the sleep quality and how comfortable they are when sleeping in bed. The longer the period of uninterrupted sleep, the more likely it was deep sleep and therefore beneficial for well-being or, in the case of subjects with COPD, that they were comfortable in bed. Although *Figure 19* does not show any specific pattern, it clearly shows that bed occupancy drops drastically between days 80 and 90, and after day 125 due to the deterioration in the condition of the patient. As we do not have full data for the PIR in the living room, we are unable to determine whether they slept in a chair in preference. However, the change in habit remains significant.

## Discussion

We have presented results from one of the first projects to deploy both environmental and physiological sensors to patients. Our results demonstrate the feasibility of integrating both types of sensor on the same interoperable platform to support health and social care together. Simple algorithms were used to generate alerts to the clinicians on the portal to indicate those patients that may require attention, prompting intervention for patients with high BP with medication change, COPD patients with low SpO_2_ for referral for pulmonary assessment and O_2_ therapy.

The habits data were capable of generating alerts for events such as a patient who had fallen, and patients with increased level of nighttime activity due to a heavy cold or exacerbation. None of these could have been identified without the integrated platform, unless the patients contacted the professionals with relevant complaints.

We have estimated habits patterns for patients, and detected deviations from normal behavior, which includes under- or overactivity alerts. Both under- and overactivity provided important information about the well-being of a subject. For example, patient 1 spent most of the day in the bedroom when they felt unwell; a sudden drop in activity level in patient 2 was found to be due to a fall and cellulitis, and later due to a leg ulcer. Previous telemonitoring studies have reported that changes in activity levels can relate to changes in well-being.^11^ For example, sleeping in a chair instead of the bed was observed with one CHF patient; frequent bathroom visits due to urinary tract infection ^24^; and decrease in activity levels due to increased depression level.^25^ Underactivity or longer time in bed could be due to depression, and overactivity due to discomfort or onset of dementia.

Our selection of features would concur well with others,^26^ who used regression analysis to determine the correlation between features of activities of daily living and self-administered health metric scores. However, in contrast, the study investigated only predictions of long-term changes in health rather than the short-term prediction of exacerbation as in this study.^26^ Neither did the study include independent clinical information or physiological data to corroborate.

We also observed association between habits data and vital signs of patients. For example, for one patient, bed occupancy dropped significantly when SpO_2_ levels fell below 85%; we believe this may be due to discomfort from dyspnea. For another patient, although we observed a slight increase in all day activity level, there was an increase in the duration of periods of inactivity. The reason for the increase in periods of inactivity was thought to be the unwillingness to move due to decreased exercise capacity or dyspnea. The increase in activity levels may be due to a slower walking pace or having to stop to catch their breath during exacerbation^21,27^ which may have resulted in two sensor events instead of one during the course of the completion of a task. These hypotheses could be tested in future studies by using accelerometers or position technology, and PIR sensors, to determine walking speed. Future studies should also collect symptoms in addition to vital signs, both of which are useful for evaluating risk of future exacerbations.^28^

Our previous work shows that changes in long-term vital signs data may have prognostic value and could be used to determine where there is need for intervention.^23^ In this study, only BP and SpO_2_ had useful information: low values of SpO_2_ led to referral for pulmonary assessment and O_2_ therapy; high values of BP, or low values when accompanied by dizziness, led to medication change and diagnosis of other conditions, such as AF. For some, the medication change was successful in establishing the desired level of BP, but for others the BP values continued to vary outside thresholds, requiring further medication change. We also noted that some BP and SpO_2_ records fluctuated with a periodic form; for example, the systolic BP for patient 1 varied between 120 and 150 mmHg with a period of 3 months.

It is clear that long-term vital signs data can provide information on the progress of the condition of a patient; however, there is currently a lack of well-defined procedures regarding how to deal with the long-term changes and trends in data, and this undermines the prognostic value of such data. For example, we observed clear indication of the progress of illness in patients with COPD in the long-term SpO_2_ readings.^23^ There is a need to determine approaches and gain knowledge to better use the long-term data to understand and manage the condition of patients. Without such approaches, effective use of all the information that is available from vital signs data is lost, and clinical trials to determine the effectiveness of telemonitoring systems are misleading, as not all the available information is being utilized.

However, we have seen that the number of clinical events in our patient population is small and so any approach to determine the effectiveness of the use of long-term vital signs data will require large, long-term observational studies.

In general, patients were very compliant and satisfied with use of the system. However, due to safety concerns regards balancing on the weigh scales, patients with CHF who had scored highly on the frailty scale did not weigh themselves often enough for reliable use of the alert algorithm or to enable management of their condition. In future studies it would be advisable to select weigh scales that are more appropriate for frail patients, such as including grab-on handles. It may also be necessary to collect other vital signs in addition to weight to have a better picture of a patient with CHF; this might include ECG, SpO_2_, and BP.^29^

### Strengths

Due to the ease of use and unobtrusive features of the platform, we managed to collect telemonitoring data for longer than a year for most patients, which provided us with a significant amount of data and experience to understand: what was a useful set sensors; best sensor locations; issues on user acceptance; what works and what does not with elderly frail subjects; and the areas that can be improved.

The main benefit of the design of the technology was that it was easy to install and use (reduced training for patient); required no user interface (reduced complexity of use and increased acceptance); no (or very low) maintenance (reduced resource requirement from the service); self-contained (did not require broadband so could be installed in any home); and unobtrusive—patients could use the devices anywhere in the home (reduced stigmatization for the patient). The platform and devices worked seamlessly: the patients were measuring their vital signs as normal without need for additional steps (such as entering the reading in a logbook or website, or having to go to a base unit to take measurements); the technology was present but not noticeable or unduly disturbing to their daily routine. These factors improved overall usability and resulted in patients accepting a monitoring period for longer than 1 year. However, there were technical issues at the beginning of the monitoring period, primarily related to the bed sensor, due to discomfort of the sensor under the mattress and the high rate of false alerts. The nurses adjusted their response to the alerts and would only take action following several consecutive alerts of the same type.

The potential for integrated telemonitoring platforms with reliable alerts is significant. The advantages of remote patient monitoring for reducing hospitalization and well-being of the patient are well documented^3^; however, this work demonstrates that habits monitoring may provide as valuable information on detection of exacerbation as monitoring vital signs, so that the two may complement detection, and together may increase the accuracy of prediction.

### Challenges

The high rate of false alarms is an issue for many telemonitoring systems^23^ and it is essential that reliable alarm algorithms are developed. However, development of such algorithms for habits data in particular, has been challenging for many reasons including type and number of sensors used; location of the sensors; house layout; and the presence of visitors. Developing robust algorithms and metrics that work reliably and effectively in the various settings and conditions is necessary for systems to be usable and deployed at scale. In addition, having multiple sensors and applying combined decision rules that use multiple parameters can further eliminate issues and reduce the rate of false alerts.

There is also a need to understand how best to analyze the habits data and present information to the professionals in a useful and manageable way. Recommendations for improved presentation of the data on the portal were made by the health professionals, which included the ability to view different levels of analysis of the data.^30^ For example, having seen an alert for habits on the portal, the health professionals wanted to see further details by clicking on a link, including a figure illustrating the long-term data (*Figs. 5–7*), hourly movement counts for each hour from all sensors (*Figs. 3* *and* *9*) and bed-time routines (*Figs. 4* *and* *15*) if possible.

There is a tendency for the attention of the clinician to be drawn to the clinical data rather than the habits data. Habits data can easily be ignored by the clinicians, especially when there is no reliable algorithm and the worth of the data is yet to be proven. This may undermine the effectiveness of habits monitoring. On the other hand, habits data may be of interest to a close relative or care provider, so that they might, for example, be reassured whether the patient is up and about.

The position of the gateway to ensure good signal to all devices was problematic in some homes. In such cases, a signal strength meter was used to identify appropriate locations, or repeaters installed to extend range.

### Limitations

One of the limitations of this study is that we were unable to account for confounding factors and bias due to patient selection; doctors might have chosen subjects with severe conditions or at a late stage of the disease so that deterioration over short monitoring periods could be observed or intervention could take place. On the other hand, telemonitoring services appear to be more effective and beneficial for patients whose disease is at an advanced stage, as they are more likely to suffer from severe adverse events and they may need medical and social interventions^7^; this was the reason why the inCASA project focused on frail elderly with at least one chronic disease(s), and why many telemonitoring projects focus on these groups.^2,31,32^

### Recommendations

Based on the challenges faced and lessons learned from our experience we are able to make some recommendations.

It is advantageous to have several PIR sensors; their location around the house would be, in descending order of importance: (1) living room, (2) bedroom, (3) bathroom, and (4) kitchen. PIR sensors in these locations not only monitor movements in the house, but can determine bathroom use and meal preparation.

A reliable bed sensor can provide vital information on the well-being of the subject, including bedtimes and bed occupancy. For example, bed occupancy results for a patient with COPD showed clear indication of the struggle to stay in bed when the condition was deteriorating (see patient 4). The bed sensors need to be designed to be comfortable and reliable, and to sense presence over a larger area of the bed than the pressure sensor used in this project.

## Conclusion

We have collected and analyzed the data from combined habits and health monitoring of 36 frail elderly participants. We have detected deviations from their normal activity profile and in the physiological data. Long-term changes in activity profile and bed occupancy were associated with the condition of the patient. For example, from changes such as bedtimes and time between activities, we could clearly observe progress of the condition and response to intervention in patients with COPD and CHF. This association between the clinical condition of patients and their behavioral data is promising, but needs to be verified with a large study.

Although BP and SpO_2_ readings were found to be very useful, simple thresholds were problematic in generating too many false alerts, and the prognostic value of these can be improved with improved algorithms and well-defined protocols on how to deal with long-term data.

Our results also showed the importance of having a simple and unobtrusive telemonitoring platform and devices for use by frail elderly patients to achieve prolonged monitoring periods and acceptance.
